# A combination of left ventricular noncompaction and double orifice mitral valve

**DOI:** 10.1186/1476-7120-7-11

**Published:** 2009-03-09

**Authors:** Xing-Xiang Wang, Ze-Zhou Song

**Affiliations:** 1 Department of Cardiovascular Medicine, The First Affiliated Hospital, College of Medicine, Zhejiang University, Hangzhou, PR China; 2 Department of Ultrasound, The First Affiliated Hospital, College of Medicine, Zhejiang University, Hangzhou, PR China

## Abstract

A 24-year-old woman admitted with mild chest distress associated with activity without chest complaint for twenty days. Two orifices were visible at the level of the mitral valve with a transthoracic short-axis view of the two-dimensional and three-dimensional echocardiography. The left ventricle was mildly dilatated and the left ventricular wall was thickened, especially at the apex and anterolateral wall, and appeared sponge-like. There were numerous, excessively prominent trabeculations associated with intertrabecular recesses. Although the coexistence of NVM and DOMV could be a coincidence, we believe that both defects were probably caused by a developmental arrest of the left ventricular myocardium in the present case.

## Introduction

Noncompaction of ventricular myocardium (NVM) is increasingly recognized as an important cause of cardiomyopathy that is thought to be related to an arrest of in normal endomyocardial embryogenesis, resulting in persistence of multiple prominent ventricular trabeculations with deep intertrabecular recesses [1]. During the second month of embryonal life, two parallel processes normally occur: (a) gradual compaction of the ventricular myocardium with transformation of the intertrabecular spaces into capillaries, and (b) development of the coronary circulation. The process of compaction progresses from the epicardium to the endocardium and from the base of the heart toward the apex. It may occur as an isolated cardiac malformation [1], and its classification as a distinct entity within the group of cardiomyopathies has been proposed recently [2]33. However, a similar persistence of NVM may be associated with congenital heart disease and it has been also reported of the possibility that NVM could present as an acquired disease [3].

Double orifice mitral valve (DOMV) is a rare congenital anomaly and it may be coincidentally diagnosed on echocardiography. However, the clinical spectrum is still obscure because of its rarity. DOMV rarely occurs as an isolated anomaly [4], but it can be associated with a variety of other cardiac anomalies such as truncus arteriosus, VSD, pulmonary stenosis, coarctation of the aorta, bicuspid aortic valve, tetralogy of Fallot, Ebstein's anomaly, and, most commonly, atrioventricular canal defect [5-9].

Left ventricular NVM associated with DOMV is a very rare pathology although this association has been reported previously [10-13]. Therefore, we present a case of left ventricular NVM associated with DOMV in an adult woman, which was confirmed by transthoracic two-dimensional and three-dimensional echocardiography, and discuss the possible mechanism of the association.

## Case report

The patient was a 24-year-old woman admitted with mild chest distress associated with activity without chest complaint for twenty days. In past, she was never found to have heart diseases, lung disease, relevant history of familial heart diseases or sudden cardiac death, hypertension, diabetes mellitus and denied relevant history of smoking.

On examination, her blood pressure was 130/75 mmHg; pulse 118 beat per minute. The electrocardiogram showed a left ventricular hypertrophy pattern with sinus tachycardia. On auscultation, a systolic murmur, grade 4/6, was audible at the left sternal border and apex. Chest x-ray revealed the heart to be mildly enlarged and 0.66 in cardiothoracic ratio, but there was no pulmonary congestion.

**Figure 1:**
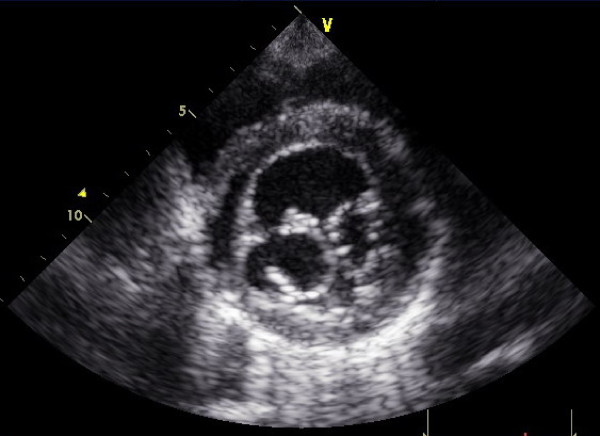
**Parasternal short-axis view shows double-orifice mitral valve by two-dimensional echocardiography**.

**Figure 2:**
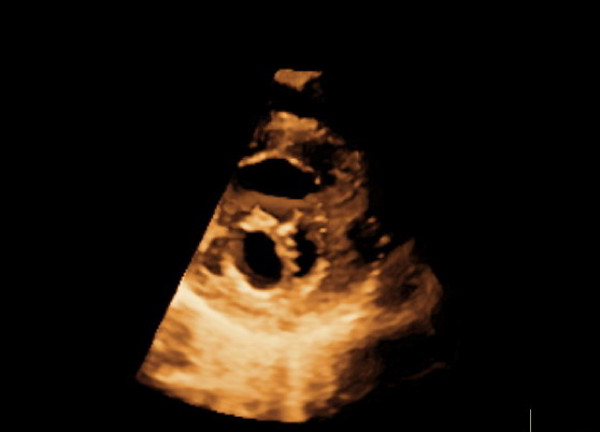
**Parasternal short-axis view shows double-orifice mitral valve by three-dimensional echocardiography**.

**Figure 3:**
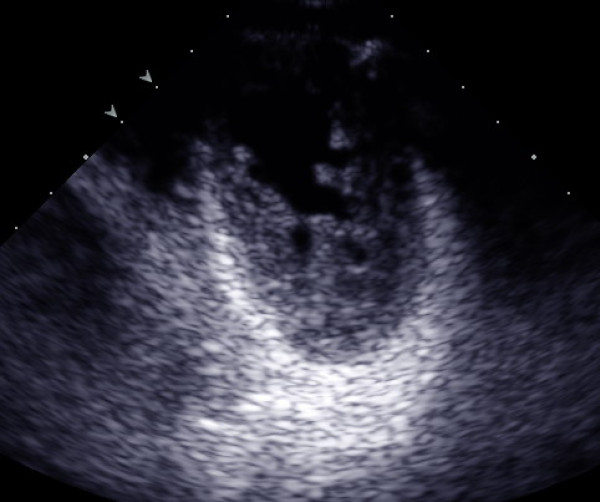
**Apical short-axis view shows numerous, excessively prominent trabeculations associated with intertrabecular recesses by two-dimensional echocardiography**.

Two orifices were visible at the level of the mitral valve with a transthoracic short-axis view of the two-dimensional and three-dimensional echocardiography (Figure 1 and 2). A mild mitral regurgitation of both orifices was obtained. The left ventricle was mildly dilatated and the left ventricular wall was thickened, especially at the apex and anterolateral wall, and appeared sponge-like. There were numerous, excessively prominent trabeculations associated with intertrabecular recesses (Figure 3). The color flow entered into the intertrabecular recesses. We diagnosed noncompaction of the left ventricular myocardium.

## Discussion

We should first ask whether left ventricular NVM could be the consequence of DOMV. In the other words, could left ventricular NVM present as an acquired disease caused by DOMV?

The cause and pathogenesis of acquired left ventricular NVM remains unknown, but several speculations can be raised to explain its occurrence [14,15]:(1) left ventricular NVM represents an insufficient, compensatory attempt of hypertrophy of the impaired left ventricular myocardium.(2) left ventricular NVM results from an attempt to enlarge the endocardial surface to move large stroke volumes with reduced contractility and to maintain a sufficient cardiac output/stroke volume in volume overload.(3) left ventricular NVM results from a "dissection" of the impaired myocardium because of reduced adhesion of cardiomyocytes and malfunction of gap junctions particularly at the most demanded regions of the myocardium with consecutive transformation to a meshwork of trabeculations. However, in the present case, it seems that the above-mentioned speculations can not explain its occurrence because the volume load is mild and there are not other overloads. Therefore, left ventricular NVM could not present as an acquired disease.

In addition, the question must be posed as to whether DOMV and left ventricular NVM are both the consequence of an additional undetermined cause?

The formation of the mitral valve begins in the fourth week of gestation and continues to the sixth month. The primitive mitral leaflets are formed from endomyocardial cushions, and the papillary muscle and chordae are formed by primitive trabeculation in the left ventricle and the endomyocardial cushions. The trabeculations continue to fuse until the 24th week, by which time they have formed the two distinct papillary muscles. By the sixth month, the muscular tissue of the chordae is replaced with thin, delicate collagenous tissue, completing the connection between the now well-formed papillary muscles and leaflets [11]. Similar to the tricuspid valve, the mitral valve is formed from endocardial cushion tissue and from ventricular myocardium, separated from the ventricular wall by undermining and diverticulation [13]. In the other words, the mitral valve and its apparatus originate from the endomyocardium, which is thought to be origin of noncompacted myocardium. Although the coexistence of NVM and DOMV could be a coincidence, we believe that both defects were probably caused by a developmental arrest of the left ventricular myocardium in the present case.

## Consent

Written informed consent was obtained from the patient for publication of this case report and accompanying images. A copy of the written consent is available for review by the Editor-in-Chief of this journal.

## Competing interests

The authors declare that they have no competing interests.

## Authors' contributions

XXW carried out physical examination and drafted the manuscript. ZZS carried out Echocardiographic examination and drafted the manuscript. All authors read and approved the final manuscript.

## References

[B1] Chin TK, Perloff JK, Williams RG, Jue K, Mohrmann R (1990). Isolated noncompaction of left ventricular myocardium. A study of eight cases. Circulation.

[B2] Jenni R, Oechslin E, Schneider J, Attenhofer JC, Kaufmann PA (2001). Echocardiographic and pathoanatomical characteristics of isolated left ventricular non-compaction: a step towards classification as a distinct cardiomyopathy. Heart.

[B3] Song ZZ, Ma J (2007). A rare combination of left ventricular noncompaction and a right coronary artery-to-right ventricle fistula: echocardiographic features. J Ultrasound Med.

[B4] Lee DI, Ha JW, Chung B (1984). Double orifice mitral valve. Clin Cardiol.

[B5] Banerjee A, Kohl T, Silverman NH (1995). Echocardiographic evaluation of congenital mitral valve anomalies in children. Am J Cardiol.

[B6] Yeßilbursa D, Miller A, Nanda NC (2000). Echocardiographic diagnosis of a stenotic double-orifice parachute mitral valve with a single papillary muscle. Echocardiography.

[B7] Sasaoka T, Ohguri H, Makita Y (1996). Double-orifice mitral valve in an elderly patient with tetralogy of Fallot. Jpn Heart J.

[B8] Yamaguchi M, Tachibana H, Hosokawa Y (1989). Ebstein's anomaly and partial atrioventricular canal associated with double-orifice mitral valve. J Cardiovasc Surg (Torino).

[B9] Lee CN, Danielson GK, Schaff HV (1985). Surgical treatment of double-orifice mitral valve in atrioventricular canal defects: Experience in 25 patients. J Thorac Cardiovasc Surg.

[B10] Kamei J, Nishino M, Hoshida S (2001). Double-orifice mitral valve associated with noncompaction of left ventricle. Heart.

[B11] Sugiyama H, Hoshiai M, Toda T, Nakazawa S (2006). Double-orifice mitral valve associated with noncompaction of left ventricular myocardium. Pediatr Cardiol.

[B12] Gorgulu S, Celik S, Eksik A, Tezel T (2004). Double-orifice mitral valve associated with nonisolated left ventricular noncompaction – a case report. Angiology.

[B13] Mierop LHSV, Alley AD, Kausel HW, Stranahan A (1962). The anatomy and embryology of endocardial cushion defect. J Thorac Cardiovasc Surg.

[B14] Finsterer J, Stfllberger C, Schubert B (2004). Acquired left ventricular hypertrabeculation/noncompaction in mitochondriopathy. Cardiology.

[B15] Finsterer J, Stfllberger C (2001). Spontaneous left ventricular hypertrabeculation in Dystrophin duplication based Becker muscular dystrophy. Herz.

